# Supplementary material for: Experimental band structure spectroscopy along a synthetic dimension
Source: Nat Commun. 2019 Jul 16;10:3122. doi: 10.1038/s41467-019-11117-9 (PMC6635488; doi:10.1038/s41467-019-11117-9)
Supplement: Supplementary file 1 — Supplementary Information [file 41467_2019_11117_MOESM1_ESM.pdf]

Supplementary Information for:

Experimental band structure spectroscopy along a synthetic dimension

Dutt et al.

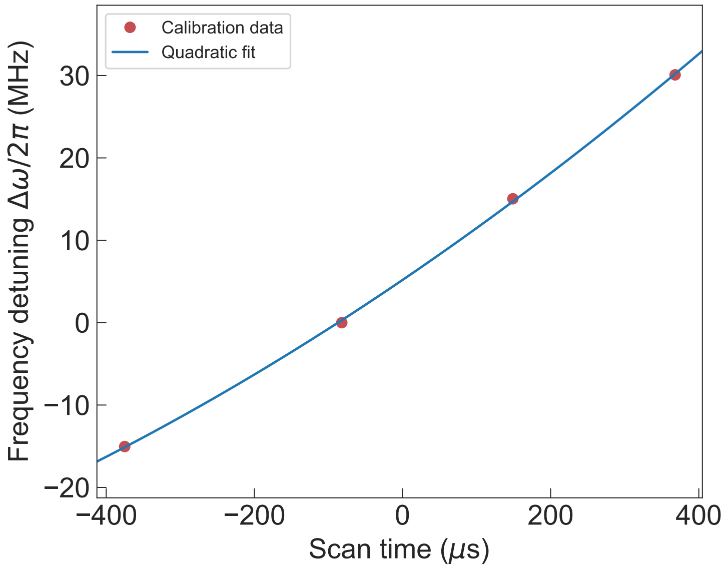

Supplementary Figure 1. Calibration of frequency detuning with time for a linear voltage ramp. The red dots are measured by noting positions of the cavity resonances, which are equally spaced by 15.04 MHz. The blue line is a quadratic fit to the measured data.

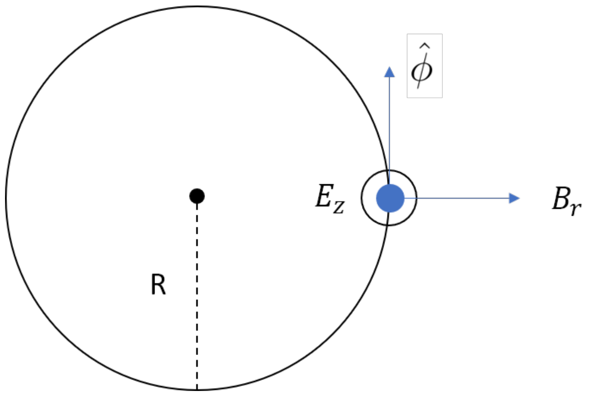

Supplementary Figure 2. A ring cavity with periodic boundary conditions.

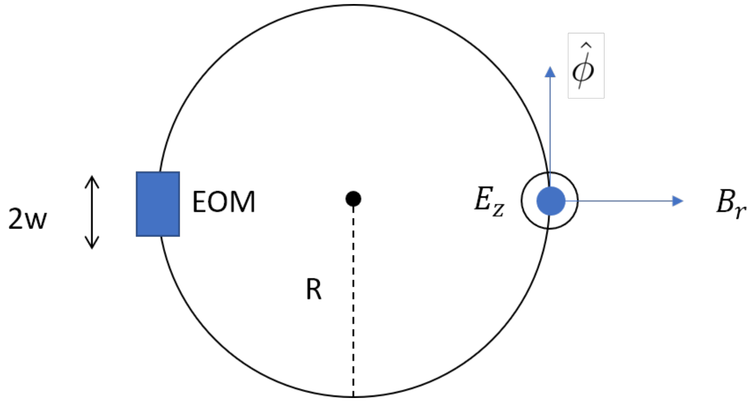

Supplementary Figure 3. A cavity with a small time modulated section.

SUPPLEMENTARY NOTE 1 - COUPLED MODE EQUATIONS FOR A MODULATED RING

In this section we derive the coupled mode equations for a ring resonator with a portion of the ring's refractive index modulated in time, say, using a phase modulator.

We consider a ring cavity where the dimensions are such that the variation only along the propagation direction plays an important role. An example would be a ring as shown in Supplementary Figure 2 whose radius is much larger than the cross sectional width of the waveguide forming the ring. The Maxwell's equations for this system then read:

$$\nabla \times \mathbf{E} = -\frac{\partial \mathbf{B}}{\partial t} \tag{1}$$

$$|\mathbf{B}| = \mu|\mathbf{H}| = \mu\frac{|\mathbf{E}|}{\sqrt{\mu/\epsilon}} = \frac{n|\mathbf{E}|}{c} \tag{2}$$

In cylindrical polar coordinates, for fields E_z, B_r and propagation along $\hat{\phi}$ on a ring of radius R,

$$\nabla \times \mathbf{E} = \hat{r}\frac{1}{R}\frac{\partial}{\partial \phi}E_z \tag{3}$$

Substituting Supplementary Eqs. (2) and (3) into Supplementary Eq. (1),

$$\frac{1}{R}\frac{\partial E_z}{\partial \phi} = -\frac{\partial}{\partial t}\left(\frac{nE_z}{c}\right) \tag{4}$$

$$\Rightarrow \frac{c}{nR}\frac{\partial E_z}{\partial \phi} = -\frac{\partial E_z}{\partial t} - E_z\frac{1}{n}\frac{\partial n}{\partial t} \tag{5}$$

At every moment in time, $e^{-im\phi}$ forms a complete basis, so we can expand

$$E_z(\phi,t) = \sum_{m'=0}^{\infty} C_{m'} a_{m'}(t) e^{-im'\phi} + c.c. \tag{6}$$

where the prefactor C_m will be chosen so that $|a_m|^2$ represents the photon number flux in mode m for an unmodulated cavity. Substituting this in Supplementary Eq. (5), we get,

$$-\frac{c}{n(\phi,t)R}\sum_{m'} im\, C_{m'} a_{m'}(t) e^{-im'\phi} = -\sum_{m'} C_{m'} \dot{a}_{m'}(t) e^{-im'\phi} - \sum_{m'} C_{m'} a_{m'}(t) e^{-im'\phi} \frac{1}{n(\phi,t)}\frac{\partial n}{\partial t} \tag{7}$$

Applying $\int_0^{2\pi} e^{im\phi}\, d\phi/2\pi$ to both sides yields:

$$\frac{c}{2\pi R}\sum_{m'}\left[im' C_{m'}\, a_{m'}(t)\int_0^{2\pi}\frac{e^{i(m-m')\phi}}{n(\phi,t)}\,d\phi\right] = C_m \dot{a}_m(t) + \frac{1}{2\pi}\sum_{m'}\left[C_{m'} a_{m'}(t)\int_0^{2\pi}\frac{e^{i(m-m')\phi}}{n(\phi,t)}\frac{\partial n}{\partial t} d\phi\right] \tag{8}$$

or,

$$\frac{d}{dt}a_m(t) = i\sum_{m'}(\kappa_{mm'} - i\gamma_{mm'})a_{m'}(t), \tag{9}$$

where

$$\kappa_{mm'} = \frac{m'c}{2\pi R}\frac{C_{m'}}{C_m}\int_0^{2\pi}\frac{e^{i(m-m')\phi}}{n(\phi,t)}\,d\phi \tag{10}$$

$$\gamma_{mm'} = \frac{1}{2\pi}\frac{C_{m'}}{C_m}\int_0^{2\pi}\frac{e^{i(m-m')\phi}}{n(\phi,t)}\frac{\partial n}{\partial t}\,d\phi \tag{11}$$

For the remainder of this section we will ignore $\gamma_{mm'}$ since the contribution from $\partial n/\partial t$ is estimated to be negligible for the modulation frequencies and modulation depths used in the experiment.

Limiting cases

A. Constant index

If $n(\phi,t) = n_0$,

$$\kappa_{mm'} = \frac{m'c}{2\pi R\, n_0} 2\pi\delta_{mm'} = m' 2\pi\frac{c}{n_0 L}\delta_{mm'} = \omega_{m'}\delta_{mm'}, \tag{12}$$

where we recover $\omega_{m'} = m' \times 2\pi c/n_0 L$ as the frequency of the m'-th cavity resonance.

$$\Rightarrow \dot{a}_m(t) = i\omega_m a_m(t) \tag{13}$$

The unmodulated cavity mode spacing is then $\Omega_R = \omega_{m'+1} - \omega_{m'} = 2\pi c/n_0 L$. The mode spacing is equal over a range of frequencies as long as the group velocity dispersion of material and the geometric dispersion due to the cavity are negligible. Since the energy of a photon in mode m is $\hbar\omega_m$, and $|a_m|^2$ represents the photon number flux, we can determine the prefactor C_m to be,

$$C_m = \sqrt{\frac{\hbar\omega_m}{2\epsilon_0 n_0^2 L}} \tag{14}$$

B. Uniform time varying index

If $n(\phi, t) = n_0 + n_1(t)$, where $n_1 \ll n_0$,

$$\kappa_{mm'} = \frac{m'c}{L}\frac{2\pi\,\delta_{mm'}}{n_0 + n_1(t)} \approx \frac{2\pi m'c}{L}\frac{\delta_{mm'}}{n_0}\left(1 - \frac{n_1(t)}{n_0}\right) = \delta_{mm'}(\omega_{m'} - \omega_{m'}(t)) \tag{15}$$

$$\Rightarrow \dot{a}_m(t) = i(\omega_m - \omega_m(t))a_m(t) \tag{16}$$

C. A small portion of the ring is time-modulated

If the length of the modulated section is $2w$ [Supplementary Figure 3], then the spatiotemporal index distribution in the ring is,

$$n(\phi, t) = n_0 + n_1(t)\,[u(\phi - \phi_w/2) - u(\phi + \phi_w/2)] = n_0 + n_1(t)\,\mathrm{rect}(\phi/\phi_w) \tag{17}$$

where $u(\cdot)$ is the Heaviside theta function, $\phi_w = 2\pi \times 2w/L$ and $n_1(t) \ll n_0$ at all times t.

$$\begin{aligned}
\kappa_{mm'} &= \frac{m'c}{L}\frac{C_{m'}}{C_m}\int_0^{2\pi} \frac{e^{i(m-m')\phi}}{n_0 + n_1(t)\,\mathrm{rect}(\phi/\phi_w)}\,d\phi && (18)\\
&\approx \frac{c\sqrt{mm'}}{L}\int_0^{2\pi} \frac{e^{i(m-m')\phi}}{n_0}\left[1 - \frac{n_1(t)}{n_0}\,\mathrm{rect}(\phi/\phi_w)\right]d\phi && (19)\\
&= \frac{c\sqrt{mm'}}{n_0 L}2\pi\delta_{mm'} - \frac{c\sqrt{mm'}}{n_0 L}\frac{n_1(t)}{n_0}\int_{-\phi_w/2}^{\phi_w/2} e^{i(m-m')\phi}\,d\phi && (20)\\
&= \omega_{m'}\delta_{mm'} - \Omega_R\sqrt{mm'}\frac{\phi_w}{2\pi}\frac{n_1(t)}{n_0}\mathrm{sinc}((m-m')\phi_w/2) && (21)
\end{aligned}$$

Thus, $\kappa_{mm'}$ is nonzero for all m, m' for a point modulation.

Eqs. (10) and (21) show that the coupling coefficient depends predominantly on $m' - m$ since the prefactor $\sqrt{mm'}$ varies slowly for large m, m' and small $m' - m$. This justifies the assumption in the main text that $J_{m,m'}$ depends on $m' - m$, establishing a discrete

modal translational symmetry along the frequency axis. In our experimental setup, $m \approx n_0 L/\lambda = 1.6 \times 10^7$ and $|m' - m| \leq 20$, so the modal translational symmetry is a very good approximation.

Using $\phi_w/2\pi = 2w/L$, the coupled amplitude equations then take the form,

$$\frac{d}{dt} a_m(t) = i\omega_m a_m(t) - i \sum_{m'} \Omega_R \sqrt{mm'} \frac{2w}{L} \frac{n_1(t)}{n_0} \operatorname{sinc}\left(\frac{(m-m')w}{2\pi L}\right) a_{m'}(t) \tag{22}$$

$$\approx i\omega_m a_m(t) + i \sum_{m'} J_{m'-m}(t)\, a_{m'}(t) \tag{23}$$

which is the same as Eq. (1) in the main text.

For the specific case of a phase modulator based on a second-order nonlinear material with an electro-optic coefficient r and a thickness d, the applied voltage $V_M(t)$ is related to the index change $n_1(t)$ in the modulated section of the ring by the expression [1, 2]:

$$n_1(t) = \frac{n_0^3 r}{2d} V_M(t) \tag{24}$$

The half-wave voltage – the voltage needed to cause a phase change of π in the modulator at a wavelength λ – is [1],

$$V_\pi = \frac{\lambda d}{2wrn_0^3} \tag{25}$$

Plugging this expression for V_π in Supplementary Eq. (24), we get a more experimentally accessible relationship between $n_1(t)$ and $V_M(t)$:

$$n_1(t) = \frac{\lambda}{4w} \frac{V_M(t)}{V_\pi} \tag{26}$$

Hence, for modes around a central mode number m_0, $\Omega_R\sqrt{mm'} \approx \omega_{m_0}$, and the coupling coefficients are related by,

$$J_{m'-m_0}(t) = -\omega_{m_0} \frac{2w}{L} \frac{n_0^2 r}{2d} \operatorname{sinc}\left(\frac{(m_0-m')w}{2\pi L}\right) V_M(t) \tag{27}$$

$$= -\omega_{m0} \frac{\lambda_{m0}}{2n_0 L} \operatorname{sinc}\left(\frac{(m_0-m')w}{2\pi L}\right) \frac{V_M(t)}{V_\pi} \tag{28}$$

$$\approx -\frac{\pi c}{n_0 L} \operatorname{sinc}\left(\frac{(m_0-m')w}{2\pi L}\right) \frac{V_M(t)}{V_\pi} \tag{29}$$

$$= -\frac{\Omega_R}{2} \operatorname{sinc}\left(\frac{(m_0-m')w}{2\pi L}\right) \frac{V_M(t)}{V_\pi} \tag{30}$$

Note that this relationship and Supplementary Eq. (23) are quite general – it does not make any assumption about the periodicity of $V_M(t)$. The weak optical frequency dependence of

this coupling is now completely contained in the half-wave voltage V_π. From Supplementary Eq. (28), we can see that the coupling coefficient can approach or even exceed the FSR if the amplitude of V_M exceeds $2V_\pi$.

SUPPLEMENTARY METHODS

Details of the experimental setup

The input laser was a grade 3 Orion laser from Redfern integrated optics [3] with a linewidth of 2.8 kHz and a center wavelength of 1542.057 nm. We apply a 0.6 V_{pp} triangular ramp signal to the frequency modulation input of the laser to scan the optical frequency over ∼50 MHz. The polarization of the light circulating in the fiber ring resonator is adjusted to excite only one principal polarization mode of the ring and match it to the principal axis of the electro-optic modulator (EOM). The EOM was a lithium niobate phase modulator with a 4.7 GHz bandwidth and a 3.4 dB insertion loss. To realize a high-Q cavity, the loss from this and other components was partially compensated by the semiconductor optical amplifier (SOA) which could provide a maximum gain of more than 12 dB when driven by a laser diode controller. In our experiment, a lower gain was used to prevent lasing. A dense wavelength-division multiplexing (DWDM) filter with a center wavelength of 1542.14 nm (Channel 44) and a 26.5 GHz passband was used after the cavity for two reasons. First, it prevented spurious lasing near the peak gain wavelength of the SOA, or at wavelengths where the roundtrip loss of the cavity is lower than at the input laser wavelength. Second, it helped to filter out amplified spontaneous emission noise outside the passband of the filter. The 100 GHz passband is much wider than the 15.04 MHz FSR, allowing a large number of modes of the ring cavity to experience flat transmission through the filter. The 2×2 fiber couplers for the through and drop ports had a nominal 99:1 splitting ratio. Both the ramp signal for the frequency sweep of the laser and the modulation signal for the EOM were generated by the two outputs of a 125 Msamples/s Red Pitaya STEMlab with 14-bit resolution and 50 MHz analog bandwidth [4–6], controlled by the open-source software package PyRPL (Python Red Pitaya Lockbox) [7].

The RF signal input to the EOM was amplified using a Mini-Circuits ZHL-3A+ coaxial amplifier, which could provide a gain of 24 dB between 0.4–150 MHz. The amplification enabled us to access strong hopping coefficients exceeding the FSR, as can be seen in the

bottom panel of Fig. 3. Note that the amplitudes of the modulation signal in Fig. 3 (0–0.233 V_{pp}) are the values before the RF amplifier. We also optically and electrically amplified the output of the drop-port photodiode before sending it to an oscilloscope to achieve a high signal-to-noise for the band structure measurement.

The SOA in the cavity increases the background photon flux that is not associated with the band structure. In our case the background photon flux does not significantly hinder the band structure measurement, since we control the SOA gain to operate the cavity sufficiently below the lasing threshold such that the peaks in the time-resolved transmission measurement can be located correctly. Some of the background noise seen outside the bands in Figs. 3 and 4 is attributable to the SOA. While the SOA helps to compensate the EOM's insertion loss and achieve a high Q ¿ 600 million, the maximum achievable drop-port transmission efficiency was observed to be still limited to 5% due to intracavity losses. Attempts to increase the efficiency by increasing the SOA gain initiates lasing.

The issues discussed above are specific to our fiber-based implementation due to the substantial insertion loss of the EOM. The implementation of synthetic frequency dimension in cavity systems with low-insertion loss modulators, such as integrated lithium niobate rings, may not need an intracavity optical amplifier and hence may not suffer from the issues of background photon flux.

SUPPLEMENTARY DISCUSSION

Extension to higher dimensional lattices

There are several avenues one could pursue to extend the 1D frequency dimension lattice we have presented to a higher dimensional lattice, as mentioned in the Discussion section. Since theoretical proposals exist for all these schemes, we focus on an extended discussion of the experimental challenges here and ways to overcome them.

First, one could extend the lattice along a real spatial dimension, as in Refs 8 and 9, by evanescently coupling multiple rings. The main challenge here would be to match the resonance frequencies of all the rings in the lattice, which can be mitigated by actively locking the lengths of each ring using a feedback stabilization scheme.

Second, one could combine other internal degrees of freedom, such as the orbital angular momentum (OAM) of light [10–12], and form a lattice with one frequency dimension and one

OAM dimension [13]. Preliminary experiments along this direction by Cheng et al. [14], have demonstrated a resonator with 46 nearly degenerate Laguerre-Gauss modes. Further work is needed to couple these modes along the OAM dimension and the frequency dimension by incorporating spatial-light modulators and electro-optic phase modulators, respectively, within the resonator.

Third, one could use the Floquet dimension generated by time-periodic modulation, independent of the FSR-separated modes, as in Refs. 15 and 16. This synthetic lattice, however, is concomitant with a synthetic electric field along the Floquet dimension, so one would have to include its effects in the theoretical description and in experimental measurements.

Finally, one could use the strategies outlined in Refs. 17 and 18 to add modulations at n times the FSR ($n \gg 1$), to form an effective 2D or higher dimensional lattice, still within a single resonator. Experimentally, this is the most straightforward of the three schemes, but it is somewhat restrictive in terms of the lattice geometries that can be achieved. For example, it would involve spurious long-range coupling between the 1D strips of a 2D square lattice, and it is difficult to achieve arbitrary gauge potentials in such a system. Some of these constraints can be mitigated by coupling two or three rings with different resonance frequencies [17].

The number of lattice sites that can be coupled along the synthetic frequency dimension is ultimately limited by group velocity dispersion (GVD). For the fiber-based setup considered here, the GVD is 23 ps^2 km^{-1} at telecom wavelengths. We can estimate the change in FSR per mode based on the expression [19, 20]:

$$\Delta\text{FSR} = -\frac{2\pi}{L^2}\frac{\beta_2}{\beta_1^3} = 2\pi L \cdot (\text{FSR})^3 \cdot \beta_2 \tag{31}$$

where $\beta_2 = d^2\beta/d\omega^2$ is the GVD and $\beta_1 = n_g/c =$ is the first-order dispersion. The long resonator length of 13.5 m amounts to a very small FSR drift of $\Delta\text{FSR} = 7$ mHz per mode. Thus, we estimate $\sim 10^6$ modes to be uniformly spaced within a fraction of the 300-kHz linewidth of the cavity, before third-order dispersion starts to play a role. Practically, the bandwidth of the fiber components in the cavity, especially the bandpass filter and the SOA, will limit the number modes to ~ 2000.

SUPPLEMENTARY REFERENCES

[1] Kuhlow, B. Modulators. In Weber, H., Herziger, G. & Poprawe, R. (eds.) *Laser Fundamentals. Part 2*, 85–110 (Springer Berlin Heidelberg, 2006).

[2] Tsang, M. Cavity quantum electro-optics. *Phys. Rev. A* **81**, 063837 (2010).

[3] Numata, K., Camp, J., Krainak, M. A. & Stolpner, L. Performance of planar-waveguide external cavity laser for precision measurements. *Opt. Express* **18**, 22781–22788 (2010).

[4] Luda, M. A., Drechsler, M., Schmiegelow, C. T. & Codnia, J. Compact embedded device for lock-in measurements and experiment active control. Preprint at http://arxiv.org/abs/1811.01901 (2018).

[5] Hannig, S. *et al.* A highly stable monolithic enhancement cavity for second harmonic generation in the ultraviolet. *Rev. Sci. Instrum.* **89**, 013106 (2018).

[6] Larsen, M. V., Guo, X., Breum, C. R., Neergaard-Nielsen, J. S. & Andersen, U. L. Fiber coupled EPR-state generation using a single temporally multiplexed squeezed light source. Preprint at http://arxiv.org/abs/1812.05358 (2018).

[7] Neuhaus, L. *et al.* PyRPL (Python Red Pitaya Lockbox) — An open-source software package for FPGA-controlled quantum optics experiments. In *Proc. 2017 European Conference on Lasers and Electro-Optics and European Quantum Electronics Conference.* EA_P_8 (Optical Society of America, 2017).

[8] Yuan, L., Shi, Y. & Fan, S. Photonic gauge potential in a system with a synthetic frequency dimension. *Opt. Lett.* **41**, 741–744 (2016).

[9] Ozawa, T., Price, H. M., Goldman, N., Zilberberg, O. & Carusotto, I. Synthetic dimensions in integrated photonics: From optical isolation to four-dimensional quantum Hall physics. *Phys. Rev. A* **93**, 043827 (2016).

[10] Luo, X.-W. *et al.* Quantum simulation of 2D topological physics in a 1D array of optical cavities. *Nat. Commun.* **6**, 7704 (2015).

[11] Luo, X.-W. *et al.* Synthetic-lattice enabled all-optical devices based on orbital angular momentum of light. *Nat. Commun.* **8**, 16097 (2017).

[12] Zhou, X.-F. *et al.* Dynamically Manipulating Topological Physics and Edge Modes in a Single

Degenerate Optical Cavity. *Phys. Rev. Lett.* **118**, 083603 (2017).

[13] Yuan, L. *et al.* Photonic Gauge Potential in One Cavity with Synthetic Frequency and Orbital Angular Momentum Dimensions. *Phys. Rev. Lett.* **122**, 083903 (2019).

[14] Cheng, Z.-D. *et al.* Experimental implementation of a degenerate optical resonator supporting more than 46 Laguerre-Gaussian modes. *Appl. Phys. Lett.* **112**, 201104 (2018).

[15] Martin, I., Refael, G. & Halperin, B. Topological Frequency Conversion in Strongly Driven Quantum Systems. *Phys. Rev. X* **7**, 041008 (2017).

[16] Peterson, C. W., Benalcazar, W. A., Lin, M., Hughes, T. L. & Bahl, G. Strong nonreciprocity in modulated resonator chains through synthetic electric and magnetic fields. Preprint at http://arxiv.org/abs/1903.07408 (2019).

[17] Yuan, L., Xiao, M., Lin, Q. & Fan, S. Synthetic space with arbitrary dimensions in a few rings undergoing dynamic modulation. *Phys. Rev. B* **97**, 104105 (2018).

[18] Schwartz, A. & Fischer, B. Laser mode hyper-combs. *Opt. Express* **21**, 6196–6204 (2013).

[19] Matsko, A., Savchenkov, A., Strekalov, D., Ilchenko, V. & Maleki, L. Optical hyperparametric oscillations in a whispering-gallery-mode resonator: Threshold and phase diffusion. *Phys. Rev. A* **71**, 033804 (2005).

[20] Yang, K. Y. *et al.* Broadband dispersion-engineered microresonator on a chip. *Nat Photon* **10**, 316–320 (2016).
