# Supplementary material for: Experimental band structure spectroscopy along a synthetic dimension
Source: Nat Commun. 2019 Jul 16;10:3122. doi: 10.1038/s41467-019-11117-9 (PMC6635488; doi:10.1038/s41467-019-11117-9)
Supplement: Supplementary file 3 — Description of Additional Supplementary Files [file 41467_2019_11117_MOESM3_ESM.pdf]

Description of Additional Supplementary Files

File name: Supplementary Movie 1

Description: Video demonstrating the band structure measurement for on-resonance modulation with only nearest-neighbor hopping, as in Fig. 3b. The right panel shows the time-resolved transmission as the laser-cavity detuning is scanned. This directly maps out the band structure shown on the left.

File name: Supplementary Movie 2

Description: Video demonstrating band structure measurement in the presence of long-range hopping and synthetic gauge potentials, as in Fig. 4c. The right panel shows the time-resolved transmission as the laser-cavity detuning is scanned. This directly maps out the band structure shown on the left.
